# Resurgence risk for measles, mumps and rubella in France in 2018 and 2020

**DOI:** 10.2807/1560-7917.ES.2018.23.25.1700796

**Published:** 2018-06-21

**Authors:** Guillaume Béraud, Steven Abrams, Philippe Beutels, Benoit Dervaux, Niel Hens

**Affiliations:** 1 Médecine Interne et Maladies Infectieuses, CHU de Poitiers, Poitiers, France; 2 Lille University, EA2694 – Santé Publique: Epidémiologie et Qualité des Soins, Lille, France; 3 Interuniversity Institute for Biostatistics and statistical Bioinformatics, Hasselt University, Hasselt, Belgium; 4 Centre for Health Economics Research & Modelling Infectious Diseases (CHERMID), Vaccine & Infectious Disease Institute, University of Antwerp, Antwerp, Belgium; 5 University of New South Wales, Faculty of Medicine, School of Public Health and Community Medicine, Sydney, Australia; 6 CHU Lille, Direction de la Recherche en Santé, Lille, France

**Keywords:** measles, mumps, rubella, measles-mumps-rubella (MMR) vaccine, modelling, outbreaks

## Abstract

Large measles and mumps outbreaks recently occurred throughout Europe and the United States. **Aim**: Our aim was to estimate and map the risk of resurgence for measles, mumps and rubella in France. **Methods**: We used a multi-cohort model combining seroprevalence information, vaccine coverage and social contact data. **Results**: The overall outbreak risk for France in 2018 was highest for mumps, remained significant for measles despite a recent measles outbreak and was low for rubella. Outbreak risks were heterogeneous between departments, as the effective reproduction numbers for 2018 ranged from 1.08 to 3.66. The seroprevalence, and therefore the risk of measles and rubella infection, differed significantly between males and females. There was a lower seroprevalence, and therefore a higher risk, for males. Infants of less than 1 year would be seriously affected in a future outbreak of measles, mumps or rubella, but the highest overall caseload contribution would come from teenagers and young adults (10–25 years old). **Conclusions**: The high risk for teenagers and young adults is of concern in view of their vulnerability to more severe measles, mumps and rubella disease and complications.

## Introduction

In 2010, the World Health Organization (WHO) Regional Office for Europe set an elimination target for measles and rubella in the European Region by 2015 [1]. Continued measles outbreaks show that this goal has not been achieved. France experienced a massive measles outbreak in 2010–11, accounting for more than half of the 30,000 cases in Europe during this period [2], and numerous sporadic outbreaks are still occurring [3]. More recently, large measles and mumps outbreaks occurred throughout Europe [4,5] and the United States (US) [6]. Although insufficient vaccine coverage is an essential factor enabling the occurrence of large-scale outbreaks, a small but noticeable proportion of affected individuals were fully vaccinated [2,7], calling into question both the lifelong persistence of vaccine-induced immunity and the optimal vaccination schedule. The current vaccine is a trivalent measles, mumps and rubella (MMR) vaccine, implying that a potential risk for measles resurgence because of incomplete coverage may be associated with risks of mumps and rubella outbreaks. The measles vaccination coverage in France is among the lowest in Europe [1], and recent measles outbreaks in neighbouring countries threaten to spill over into France, thereby potentially catalysing a new European outbreak.

The measles, mumps and rubella resurgence risks were recently estimated for Belgium, a country with a highly vaccinated population and recent mumps and measles outbreaks [8-10]. Applying a similar approach, using French data on vaccine coverage and seroprevalence obtained from cross-sectional studies, we aimed at estimating and mapping the measles, mumps and rubella resurgence risks for France in 2018 and in 2020 to illustrate future trends, notably whether the 2010–11 outbreak had sufficiently ‘mopped-up’ susceptibles to safeguard France from future (major) measles outbreaks.

## Methods

### Datasets

We used two serological datasets for the years 2009 and 2013, respectively.

The 2009 serological dataset was obtained by merging two complementary seroprevalence studies in France [11]. The Saturn-Inf national study collected serum samples from 1,617 hospitalized children aged 6 months to 6 years between September 2008 and February 2009. Starting in April 2009, the Sero-Inf study included 5,300 individuals aged 6 to 49 years who visited a laboratory for a blood test in metropolitan France over a 6-month period. The two studies provided serological data on measles, mumps and rubella.

The 2013 serological data came from the Sero-RR study, which measured the seroprevalence for measles and rubella among 4,647 blood donors aged 18 to 32 years in France, during the second half of 2013.

Since 1986, the first dose of MMR vaccine has been administered to children in France at 1 year of age. From 1996 to 2005, a second MMR dose was administered to children aged 3 to 6 years, and it has been administered at 2 years of age since 2005. Vaccine coverage at 24 months was documented by department (an intermediate division of the French administrative territory) from 2004 to 2011 [12].

Social contact data were collected in 2012 within the Comes-F study [13], resulting in the provision of French contact matrices.

### Vaccine failure rates

We chose a conservative approach (i.e. minimising the outbreak risk) in which infection is assumed to confer lifelong immunity [14]. We considered seronegative individuals as fully susceptible and ignored possible cellular immunity, about which no data are available.

Newborns lose the protection provided by maternal antibodies after an average of 3 months if born to vaccinated women and 5 months if born to naturally immune women [15,16]. Therefore, we assumed that maternal antibodies decay exponentially at a rate of 3.87 year^− 1^ (i.e. the mean duration of maternal protection is 3.1 months) to account for the mix of children born to vaccinated and naturally immune mothers [8].

Primary and secondary vaccine failure rates were estimated from the literature (Supplement). Different published estimates were combined with a random-effects meta-analysis approach to calculate the overall estimates for seroconversion (primary vaccine failure) and waning rates (secondary vaccine failure) [8-10]. However, for rubella, this approach was not relevant due to the extreme heterogeneity in the very few available published studies; therefore, we estimated the waning rate using the European Sero-Epidemiology Network (ESEN) 2006 study [17], which provided seroprevalence data for 21 European countries and Australia. The seroconversion and exponential waning rates are summarised in the Table. As a sensitivity analysis, we also studied two alternate scenarios. One omitted waning, as the rubella waning rate estimated from ESEN 2006 was not significantly different from zero, and one was estimated from the literature using a fixed-effects meta-analysis, for which the waning rates may have been overestimated (Supplement).

**Table:** Estimated seroconversion rates and exponential waning rates for measles, mumps and rubella, according to the meta-analysis conducted in 2014 [8-10]

	Measles	Mumps	Rubella
**Seroconversion rates (95% CI)**			
	0.977 (0.959 to 0.990)	0.934 (0.910 to 0.954)	0.984 (0.974 to 0.992)
**Exponential waning rates (95% CI)**			
**After 1 dose**	0.007 (0.003 to 0.018)	0.043 (0.029 to 0.065)	–
**After 2 doses**	0.008 (0.004 to 0.020)	0.025 (0.015 to 0.042)	–
**Common waning rate**	0.008 (0.005 to 0.014)	0.030 (0.021 to 0.043)	0.003 (-0.023 to 0.028)

CI: confidence interval.

### The multi-cohort model

We estimated the effective reproduction numbers *R_e_*, which are the expected numbers of secondary cases generated by a single infectious case during their entire infectious period, when introduced into a partially immune population. If *R_e_* < 1, the epidemic will die out; an epidemic can only occur if *R_e_* > 1. The calculations were based on assumptions about the basic reproduction number *R*_0_, which is the same quantity as the *R_e_* in a fully susceptible population, and on the mean infectious period *D*. The assumed measles, mumps and rubella *R_0_* estimates were 12, 10 and 8 respectively, and the *D* values were 6/365 years, 6/365 years and 7/365 years, respectively [18,19]. We also provided results for larger *R_0_* values, consistent with estimates in the literature [20].

**Figure 1:**
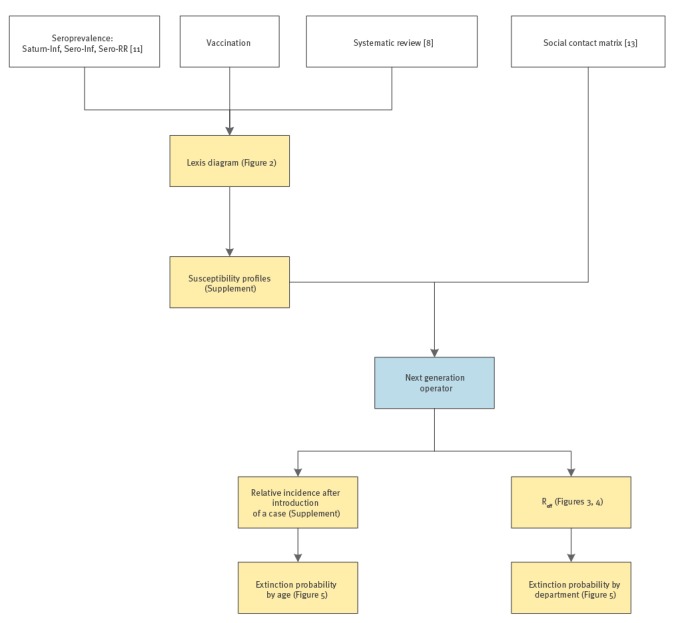
Flowchart of the general methodology of the study on the resurgence risk for measles, mumps and rubella in France in 2018 and 2020

The multi-cohort model combined serological and vaccine coverage information [8]. The general methodology is summarised in Figure 1 and detailed in the Supplement.

Items filled in yellow are related to an original result provided in the article.

**Figure 2:**
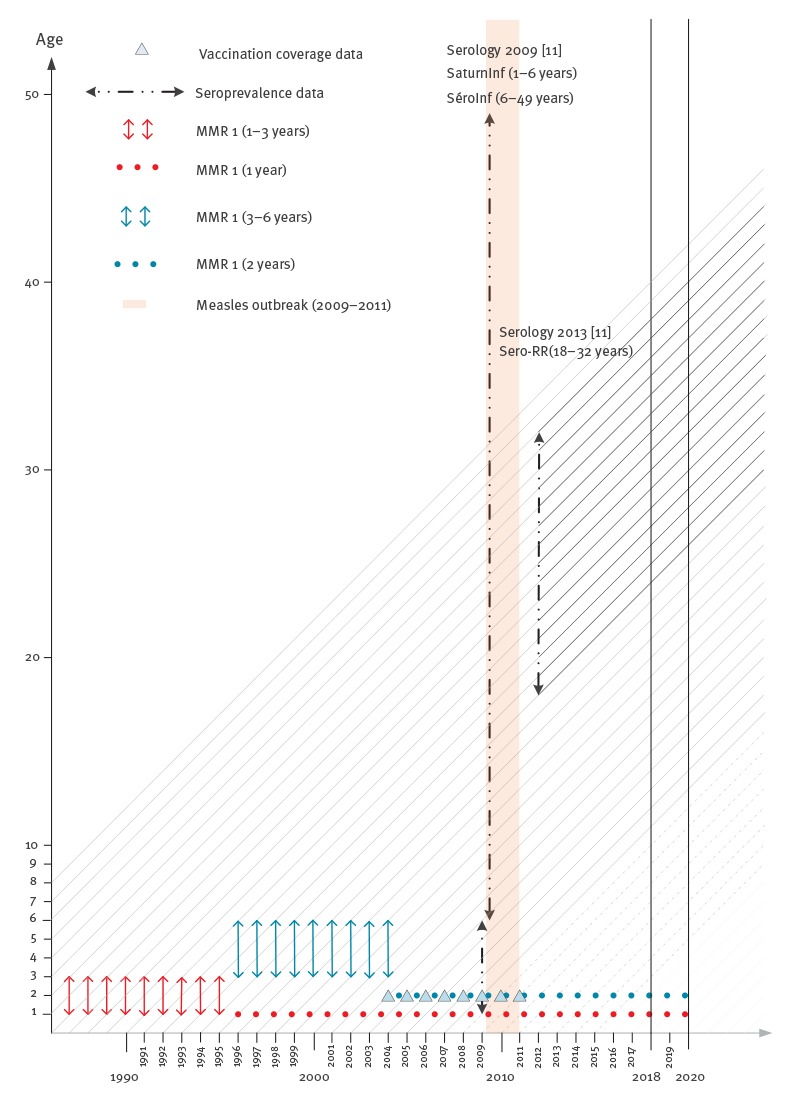
Lexis diagram showing the ageing of the yearly cohorts (1996–2023) and the period of reported cases for measles, collection times for the seroprevalence surveys (2009 and 2013), vaccination coverage information, age of vaccination and years of interest (2018 and 2020), study on the resurgence risk for measles, mumps and rubella in France in 2018 and 2020

In brief, (i) an optimal model for the serology of measles, mumps and rubella was determined for the data collection year (2009 or 2013) with the observed seroprevalence as a function of age, sex and spatial location. The best model was selected based on the Akaike Information Criteria (AIC). The mumps seroprevalence was modelled with the 2009 dataset, measles with the 2013 dataset (the 2009 dataset would not have been representative due to the 2010–2011 measles outbreak) and rubella with both; (ii) the age-dependent susceptibility by department was predicted for the years of interest (2018 and 2020), taking into account the ages and years of birth of the individuals, seroconversion rate, decay rates of vaccine-induced immunity after one and two doses, proportion of susceptible newborns (related to the proportion of susceptible women of childbearing age) and decay rate of maternal antibodies. For the youngest individuals, for whom susceptibility could not be estimated, we used the vaccine coverage and accounted for the waning of vaccine-induced immunity. In the absence of data about older ages, at which natural immunity would provide lifelong protection, we set the proportion of susceptibility equal to that of the penultimate age as determined by the seroprevalence data. The Lexis diagram (Figure 2) illustrates the use of the different datasets in the multi-cohort model; (iii) the effective reproduction number *R_e_* and the age-dependent relative incidence of a potential outbreak were estimated by department, using social contact data. These estimates are, respectively, the maximum eigenvalue and right eigenvector of the next generation operator, obtained by the product of the mean duration of infectiousness *D* with the number of susceptible individuals (the number of individuals of age *a N(a)* multiplied by the proportion of susceptible individuals of age *a*, namely the susceptibility profiles) and by transmission rates *β(a,a´)* (i.e. the per capita rate at which an infectious individual of age *a´* makes an effective contact with a susceptible individual of age *a*). An *R_e_* < 1 ensures that an incipient outbreak will die out, whereas it will spread for an *R_e_* > 1. The contact matrices were not spatially determined (i.e. there were no department-specific contact rates). However, we used sex-specific contact matrices for measles and rubella, because the susceptibility was found to be significantly different for males than for females when relying on the measles and rubella serology. Therefore, we accounted for sex differences in both susceptibility and mixing patterns, and we calculated the age-dependent relative incidence by sex.

MMR 1: first dose of measles-mumps-rubella vaccine; MMR 2: second dose of measles-mumps-rubella vaccine.

We used a parametric bootstrap with 2,000 bootstrap samples to provide 95% confidence intervals (CIs) with our estimates. Calculations were made with the R programming environment 3.1.0 [21]. The entire R-code is available upon request.

We compared the *R_e_* using a contact matrix for regular periods and holidays, simulating the occurrence of an outbreak during regular periods or school holidays [22].

Escape probabilities (the probability for an outbreak to die out from the beginning) were approximated by a branching process and derived from *R_e_* for escape probabilities by department according to three different scenarios that thereby represented various proportions of index cases [23], as well as from the age-dependant relative incidence for the escape probabilities by age [24] (Supplement). These probabilities are expressed as the probability of having a pathogen-specific outbreak (i.e. 1-escape probability).

## Results

The model selection resulted in a model accounting for sex for both measles and rubella, but not for mumps (Supplement).

**Figure 3:**
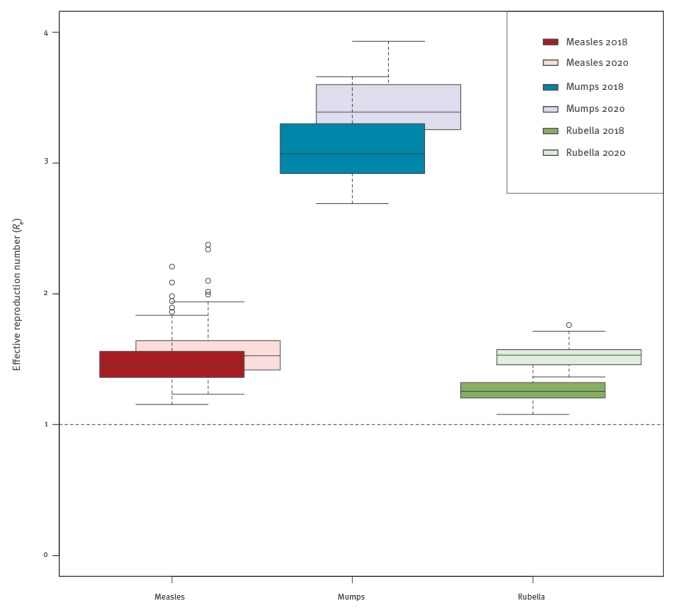
Boxplot of the effective reproduction numbers (*R_e_*) among the French departments for measles, mumps and rubella for 2018 and 2020, respectively, study on the resurgence risk for measles, mumps and rubella in France in 2018 and 2020

**Figure 4:**
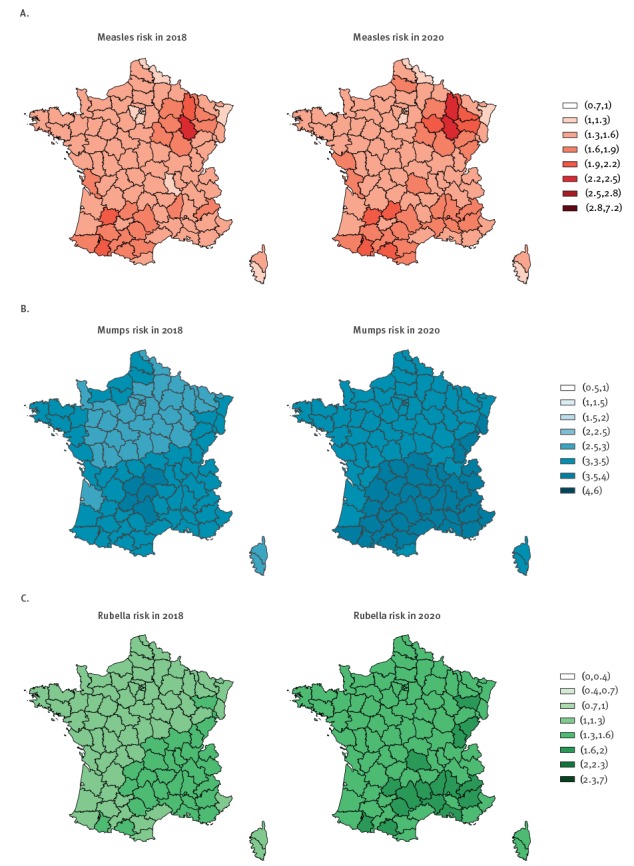
Resurgence risk maps illustrating an estimation of the effective reproduction numbers (*R_e_*) for each department for (A) measles, (B) mumps and (C) rubella for 2018 and 2020, respectively, study on the resurgence risk for measles, mumps and rubella in France in 2018 and 2020

Figure 3 represents the distribution of the effective reproduction numbers (*R_e_*) by department in France for 2018 and 2020, which are high for mumps (2018: median 3.07 (range: 2.69‒3.66); 2020: median 3.39 (range: 3.04‒3.93)), moderate for measles (2018: median 1.46 (range: 1.15‒2.21); 2020: median 1.53 (range: 1.23‒2.38)) and low for rubella (2018: median 1.25 (range: 1.08‒1.46); 2020: median 1.53 (range: 1.37‒1.76)); Figure 4 represents their spatial distribution. The *R_e_* values, average susceptibilities by age and age-dependent relative incidence (Supplement) highlight the sex differences for both measles and rubella. There are important variations between departments in the susceptibility profiles, and therefore in the outbreak risks, and in age-dependent relative incidence upon the introduction of the disease when it led to an outbreak. The curves for each department are available via the Supplement.

Males’ susceptibility is usually higher than that of their female counterparts, a finding that appears more clearly for departments with high *R_e_* estimates (Supplement).

### Measles

In 2018, the overall median Re was 1.46 (range: 1.15‒2.21) and the highest was 2.21 (95%CI: 1.06‒5.42) in Haute-Marne (department in the East of France), with an average increase of 5.0% for 2020. All departments had an Re > 1.

### Mumps

In 2018, the overall median Re was 3.07 (range: 2.69‒3.66) and the highest was 3.66 (95%CI: 3.06‒4.43) in Cantal (department in the south of France), with an average increase of 10.5% for 2020. All departments had an Re > 2 in 2018 and > 3 in 2020.

### Rubella

In 2018, the overall median *R_e_* was 1.25 (range: 1.08‒1.46) and the highest was 1.46 (95%CI: 0.82‒2.54) in Aveyron (department in the south of France), with an average increase of 20.8% for 2020. All departments had an *R_e_* > 1 in both 2018 and 2020.

The *R_e_* values in 2018 (2020) decreased during holidays, compared with regular periods, by 26.3% (26.6%), 29.2% (30.0%) and 30.0% (25.0%) for measles, mumps and rubella, respectively.

In addition to the differences between departments, some similarities arose. Infants younger than 1 year of age were likely to be highly affected by an outbreak due to the rapid waning of maternal antibodies. The highest share of cases during an outbreak would concern teenagers and young adults (10‒25 years old) with variations depending on sex, pathogen and department. Namely, in 2018, half of the measles cases would be 18 years or older for males and 19 years or older for females (19 and 20 years in 2020), half of the mumps cases would be 26 years or older (28 years in 2020) and half of the rubella cases would be 16 years or older for males and 14 years or older for females (14 and 13 years in 2020).

**Figure 5:**
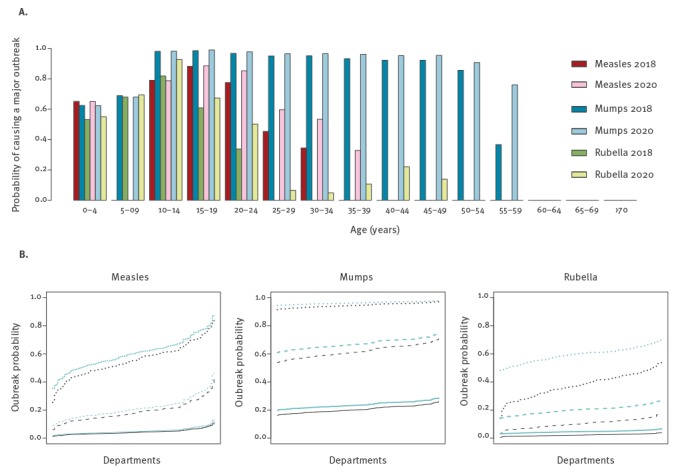
Outbreak probabilities for measles, mumps and rubella by (A) by age of index cases and (B) department, in 2018 and 2020, study on the resurgence risk for measles, mumps and rubella in France in 2018 and 2020

Figure 5 shows the outbreak probability according to the department and age of the index case, confirming the high, moderate and low risks for mumps, measles and rubella, respectively. The outbreak probability is relatively low for a young child above 1 year of age as the index case, reaches a maximum for a teenager or a young adult, and then decreases for measles and rubella, but not for mumps.

Outbreak probabilities by department (B) are presented with ratios of the variance to the mean of 1 (continuous lines, for rare super-spreading events), 5 (dashed lines, for a medium proportion of super-spreading events) and 25 (dotted lines, for numerous super-spreading events) for 2018 (black line) and 2020 (blue line).

A sensitivity analysis (Supplement) using larger *R_0_* values (16, 14 and 12 for measles, mumps and rubella, respectively) showed increases in the risk of measles, mumps and rubella outbreaks of 33.3%, 40.0% and 50.0%, respectively, but with a similar geographical distribution. Furthermore, modelling the resurgence risk for rubella using the waning rate estimated with a fixed-effect meta-analysis resulted in an *R_e_* barely higher (mean: 1.38; range: 1.16–1.54) than when the rate was estimated from the ESEN data (mean: 1.26; range: 1.08–1.46), whereas assuming a zero waning rate resulted in markedly lower risk estimates (mean: 0.57; range: 0.32–0.77). Overall, the three approaches suggested a minimal resurgence risk for rubella.

## Discussion

We estimated a re-emergence risk for measles, mumps and rubella in France, accounting for sex and spatial heterogeneity.

We showed that the risk of a new measles outbreak persists, despite the recent outbreak. In fact, 220 cases of measles were reported in Haut-Rhin (department in the Grand Est region of France) in April 2015, secondary to a school trip to Berlin where a measles outbreak was ongoing. Among the 214 cases with known vaccination statuses, 189 were not vaccinated and 22 had received only one dose. The occurrence in a department identified as being least at risk (Figure 4) could explain the rather limited expansion and largely successful containment of the outbreak. However, there are neighbouring departments close to Haut-Rhin with much greater risks of having a measles epidemic. More recently, in November 2017, a measles outbreak started in Gironde, a department in southwest France that is at much higher risk than Haut-Rhin. It resulted in a large outbreak of more than 2,000 cases all over France, which is currently fading out [25]. In addition, there are risks for mumps and rubella outbreaks, with a clearly higher risk for a mumps outbreak than for the two others. The locations of the departments most at risk for mumps and rubella largely overlap, since it concerns the south-east/south-centre area of France, where some of the lowest vaccination coverages occur for both the first and the second MMR dose. Departments at risk of measles are more scattered, probably as a consequence of the recent outbreak. The fact that departments that are at risk are not spatially clustered could limit the risk of a larger outbreak, but the current sizeable flow of long-distance travel has rendered spatial proximity relative [26]. Ultimately, our model predicts an occurrence risk for an outbreak, not an outbreak occurrence. Since the upper limit of the confidence interval of *R_e_* exceeded 1 for all the departments, an outbreak could occur anywhere. Still, estimating the amplitude of the risk helps us to identify the departments where more efforts are required to achieve effective protection. In agreement with the results for Belgium, the resurgence risk persists in the highly vaccinated French population, despite a recent substantial outbreak yielding a decrease in susceptibility.

The higher risk of a mumps outbreak results from a less effective vaccine than those for measles and rubella (Table, Supplement), from the absence of a recent outbreak that would have increased the proportion of mumps-seropositive individuals and, obviously, from a high infectiousness level as reflected by a high value for the basic reproduction number *R_0_*. By contrast, the risk of a rubella outbreak is lower due to a lower infectiousness and a very effective vaccine component (waning was not significantly different from zero, according to both the ESEN data and the meta-analysis of the literature). Despite the increased proportion of measles seropositivity resulting from the recent measles outbreak, there is a persistent risk of re-emergence due to high infectiousness.

The predominant risk of resurgence in France primarily concerns mumps, remains broadly present for measles and is minimal for rubella. In addition, we showed that the risk will increase from 2018 to 2020, and it will indeed continue to increase over the following years for the three diseases [8,9] unless a major outbreak occurs or a catch-up vaccination campaign is successfully implemented to compensate for the waning of vaccine-induced immunity and insufficient coverage.

We showed a high risk not only for infants aged less than 1 year of age, but also for teenagers and young adults for the three diseases. This finding is of paramount importance because complications are more frequent and more severe for these age groups. Whereas the mean age of onset for these infections was between 5 and 6 years during the pre-vaccine era, implying a lower risk of complications, it was 16 years (interquartile range: 7–24 years) during the last French measles outbreak [2], consistent with our projections on the median age of onset in a future outbreak. Therefore, the common knowledge indicating that measles, mumps and rubella are considered as benign diseases dates back to the pre-vaccine area and is not valid anymore. In addition to the known complications of measles, some studies showed that measles induced an immunomodulation, predisposing patients to opportunistic infections for as long as 2 to 3 years [27,28]. Therefore, the influence of measles on mortality and morbidity goes beyond the direct mortality and morbidity usually attributed to measles.

Our results suggest that a measles, mumps or rubella outbreak has less chance of spreading if it occurs on school holidays rather than during regular periods. This phenomenon has also been previously found for influenza [23,29,30].The use of empirical social contact data during holidays as a proxy for school closures [22] provides a way to estimate the impact of school closures on an incipient outbreak. Furthermore, it does not rely on a specific pathogen or context except for its transmission route. In practice, school closures would have to be timely set to be effective [31], and they would have a social and macroeconomic cost that could make them politically infeasible [32].

The probability of an outbreak (Figure 5) as the chance for an outbreak to not die out early (Supplement) after the introduction of a single case of infection highlights the participation of teenagers and young adults in the spread of the outbreak. However, the introduction of more than one infected individual will increase these outbreak probabilities. As an example, the introduction of 10 measles-infected individuals to Haut-Rhin in 2018 would result in an outbreak probability of 80.3% (1–0.850^10^) instead of 15.0% (1–0.850) with a single individual (and a variance-to-mean ratio of 5).

Therefore, the most efficient and feasible interventions to limit the risk of an outbreak would need to be focused on improving vaccination coverage. The coverage for MMR vaccine at 24 months among children born in 2010 was 91.4% for the first dose and 62.8% for the second dose [12], and it is probable that there was higher coverage among girls [33]. Accordingly, the susceptibility assessed by the seroprevalence studies was usually higher among males than females, which could be partially related to the initial use of the monovalent rubella vaccine and subsequent MMR vaccine, specifically among teenage girls to protect them from rubella [34]. Sex-specific differences in susceptibility highlight the relevance of considering sex-specific vaccination strategies. Improving vaccination coverage among girls could be preferred because they have more contacts [13] and may, therefore, be more likely to spread the virus. Improving vaccination coverage among boys could be easier because of a larger margin for improvement. However, the theoretical benefit of sex-specific vaccination strategies may be counterbalanced by their suboptimal acceptability by the general population or a negative effect on vaccination coverage, and they could, therefore, be globally counterproductive.

Our findings also highlight the need for better serological data, as data sparseness in some departments resulted in wide CIs. Moreover, the interpretations of seropositivity as a proxy for protection and seronegativity as a proxy for susceptibility have been questioned. The mumps antibody levels among students involved in a mumps outbreak at Kansas University revealed that affected individuals had lower titres than exposed subjects who did not develop mumps. However, the titres overlapped and the statistically determined cut-off values did not distinguish all the cases from the non-cases [35]. Additionally, two fully vaccinated physicians who were infected by patients with measles developed an atypical and mild form of measles diagnosed a posteriori, and did not transmit the disease despite providing care to more than 100 patients [36]; likewise, six twice-vaccinated healthcare workers contracted measles from patients but developed mild symptoms without transmitting the disease [37]. Therefore, the probabilities of being infected after contact, and of transmitting the disease, could be correlated not only with being above or below a threshold but also with the level of antibodies. In our study, we only considered seropositivity to occur when the titres were above the defined threshold; patients with a positive serology (i.e. detected antibodies), but with titres below the threshold, were considered as seronegative. One improvement would be to use a probability distribution of being infected and/or transmitting the disease as a function of the antibody level [38]. However, these data are currently unavailable. This consideration highlights the difficulty of identifying a proxy for immunity based on the antibody levels.

The selected model included sex for rubella and measles but not mumps. While unsurprising for rubella, this finding was unexpected for measles, although it was consistent with the literature on the impact of sex on measles immunology [39], incidence [40] and transmission [41]. If the significant difference between males and females for measles resulted only from the overuse of MMR vaccines among girls, it should also have been significant for mumps. Therefore, the selection of a model for mumps without considering sex is surprising. This result could be due not only to the reduced effectiveness of the vaccine compared with the vaccines against measles and rubella, which blurs the differences in natural immunity and eventually vaccination coverage, but also to the data, which might not be precise enough to express such a difference.

Our study has several limitations. French guidelines recommend a catch-up dose of MMR for unvaccinated children between 6 and 13 years of age. Although poorly applied, we lack precise information on the coverage of this catch-up dose. Therefore, we could not take it into account, albeit this dose could lower the risk of an outbreak. But this limitation concerns only data from 2013 (measles and partially rubella), as we had to rely on the available vaccine coverage information for these age categories. Another limitation is that the 2010 outbreak resulted in a temporary increase in vaccination, which may have lowered susceptibility and consequently the risk of resurgence. This possible influence exclusively concerns mumps risk modelling for which the data were related to 2009, and this ‘outbreak inspired catch-up’ concerned teenagers and young adults. Measuring seroprevalence among hospitalised patients and blood donors (i.e. individuals with potentially better access to healthcare, hence better vaccine coverage) may have minimised the resurgence risk. This would not change our conclusion, as our estimates are already noticeably high.

We adopted a conservative approach by not choosing the highest estimations of *R_0_*, but we provided a sensitivity analysis showing the increased risk of an outbreak resulting from higher *R_0_* values, which does not affect our primary conclusions.

In conclusion, we estimated a persistent high resurgence risk for mumps and measles and a relatively lower risk for rubella. This risk varies by department and sex. In addition to young infants, primarily teenagers and young adults would be affected by these outbreaks. As part of the efforts to improve vaccination coverage, the public perception of measles, mumps and rubella as generally harmless diseases should be addressed to prevent future outbreaks of these vaccine-preventable diseases.
